# Development of Non-Profit Organisations Providing Health and Social Services in Rural South Africa: A Three-Year Longitudinal Study

**DOI:** 10.1371/journal.pone.0083861

**Published:** 2013-12-16

**Authors:** Mosa Moshabela, Shira Gitomer, Bongiwe Qhibi, Helen Schneider

**Affiliations:** 1 School of Public Health, University of Witwatersrand, Johannesburg, South Africa; 2 Rural AIDS and Development Action Research, University of Witwatersrand, Acornhoek, South Africa; 3 Geneva Global, Wayne, Philadelphia, Pennsylvania, United States of America; 4 School of Public Health, University of the Western Cape, Cape Town, South Africa; Vanderbilt University, United States of America

## Abstract

**Introduction:**

In an effort to increase understanding of formation of the community and home-based care economy in South Africa, we investigated the origin and development of non-profit organisations (NPOs) providing home- and community-based care for health and social services in a remote rural area of South Africa.

**Methods:**

Over a three-year period (2010-12), we identified and tracked all NPOs providing health care and social services in Bushbuckridge sub-district through the use of local government records, snowballing techniques, and attendance at NPO networking meetings—recording both existing and new NPOs. NPO founders and managers were interviewed in face-to-face in-depth interviews, and their organisational records were reviewed.

**Results:**

Forty-seven NPOs were formed prior to the study period, and 14 during the study period – six in 2010, six in 2011 and two in 2012, while four ceased operation, representing a 22% growth in the number of NPOs during the study period. Histories of NPOs showed a steady rise in the NPO formation over a 20-year period, from one (1991-1995) to 12 (1996-2000), 16 (2001-2005) and 24 (2006-2010) new organisations formed in each period. Furthermore, the histories of formation revealed three predominant milestones – loose association, formal formation and finally registration. Just over one quarter (28%) of NPOs emerged from a long-standing community based programme of ‘care groups’ of women. Founders of NPOs were mostly women (62%), with either a religious motivation or a nursing background, but occasionally had an entrepreneurial profile.

**Conclusion:**

We observed rapid growth of the NPO sector providing community based health and social services. Women dominated the rural NPO sector, which is being seen as creating occupation and employment opportunities. The implications of this growth in the NPO sector providing community based health and social services needs to be further explored and suggests the need for greater coordination and possibly regulation.

## Introduction

Globally, lay health workers (LHWs) are increasingly recognised as essential to the health care system [1–3]. Currently, there is renewed interest in the services of LHWs, recently sparked by the need to effectively respond to HIV/AIDS amidst weakened health systems in resource-poor settings [4–7]. The current wave of research on LHWs is largely focused on the impact made by the work of LHWs on the outcomes of illness and their interventions [8–11]. In addition, a growing body of work has documented characteristics and experiences of LHWs, particularly their profiles, motivators, and risks [4,7,12]. There is, however, less clarity on the organisational aspects involving the work of LHWs. LHWs exist along a continuum of formalisation and integration with the health sector [1]. Roles vary from specialist — such as facility based antiretroviral therapy (ART) or tuberculosis (TB) counsellors, to ‘generalists’—those who work in the community conducting home visits to address a wide array of needs, often affiliated with local non-profit organisations (NPOs). The two groups of LHWs are often considered volunteers, based on the fact that they are not formally employed [11–13]. However, when funds are available, they tend to receive a small stipend [14]. The current study is concerned with NPOs implementing health and social services in rural South Africa. The NPOs operate with a cadre of LHWs known as community care workers (CCWs), who function as volunteer generalists in the provision of home-based care services.

The funds for payment of LHW stipends are often channelled through the NPOs. These NPOs can be classified broadly according to three levels of organization [15]. First, there are small community-based organisations (CBOs) organized at a local grassroots level and led by local members of the community. Second, there are larger non-governmental organisations (NGOs) operating at a provincial or national level, often sub-contracting CBOs to carry out their mandates. Lastly, there are international agencies operating in several countries, usually associated with humanitarian missions. As opposed to urban-based NPOs, rural NPOs in South Africa are largely of CBO nature, are often poorly-resourced and fragmented in social network structure, and tend to offer home-based care services and fewer community development or mobilisation activities [16]. As early as 1989, there was already recognition by Bratton that NPOs are mushrooming in resource-poor settings [15]. Since then, LHW programmes have been on the rise [1], and the NPO models of care are increasingly gaining attention in research [17]. However, a recent scoping review confirmed that literature on CBOs in low and middle-income countries is very scarce [18]. Much uncertainty exists as to the size and nature of the NPO sector in resource-limited settings, a problem that is said to have hampered policy development in South Africa [1,16]. These uncertainties exist in South Africa despite governance of the NPO sector by the Non-Profit Organisations Act No. 71 of 1997, which was intended to create enabling legal and fiscal environments. Furthermore, these uncertainties may also explain the continued marginalization of the NPO sector by government and in policy processes. In light of this gap, we investigated the origin and formation of NPOs for health and social services in rural South Africa. In particular, we sought to answer the following questions: 1) How many NPOs exist in the study area? 2) Who are the founders? 3) How and why are NPOs formed? The study aims to enrich our contextual understanding of the landscape in which LHW programmes are designed, organized, and implemented.

## Methods

### Design

In order to address the study questions, we conducted a longitudinal exploratory study of all known NPOs in a well-demarcated study area, which were interviewed annually over the three-year study period from 2010 to 2012. A descriptive approach was chosen to cater to the formative and exploratory nature of the study. The research was part of the larger project known as Care in the Home Study (CHoS), which was set up to investigate the quality of care provided by caregivers in the households of a rural South African setting.

### Setting

The rural study setting of the Bushbuckridge Local Municipality is home to nearly 542,000 people, according to the 2011 national census. In the public health sector, three hospitals, two health centres, and 37 clinics serve the population. The area is characterised by high rates of unemployment and under-development. Over half of the households earn less than ZAR800 (US$  90) per month, and approximately 80% of people are unemployed. Furthermore, the area has been designated as one of the 22 most poverty-stricken areas and one of the 13 high priority development zones. Circular labour migration to big cities is common due to the remote location of Bushbuckridge, including seasonal migration to farms. However, there is a high tourist passage through the area due to its proximity to private game lodges and the Kruger National Park.

### Sampling and Participants

We sought to identify and include all NPOs that provide health and/or social services through community-based activities and in households, and through the use of CCWs. At community-level, NPOs were referred to as community “drop-in centres”, as home-based care organizations or simply NGOs. As a starting point, a list of known NPOs was obtained from local government sources. Additional NPOs were identified from a monthly meeting forum of NPOs in Bushbuckridge. Contact details for other NPOs were provided by word-of-mouth, following clues provided by known NPOs when prompted in a form of snowball technique. NPOs that did not provide health or social services were excluded from the study. All NPOs were informed beforehand about the three-year project, and appointments were made with each NPO for the interview. 

### Data Collection

Two field workers trained by the research team conducted face-to-face interviews with managers, founders, directors, or other senior management staff of the NPOs. In 2010, 2011, and 2012, open-ended questions were used to obtain information from newly identified NPOs on the origin and status of the NPO, including information on background of the founder, reason for and process of starting the NPO, further development, challenges encountered, and future plans for the NPOs. Clarification and probing were used to encourage participants to deepen the level of description in their responses. In 2011 and 2012, follow up interviews were conducted with NPOs that were previously interviewed, concentrating on the change in status since the last interview, new information not previously reported, and verification of previously provided information. In these cases, a general interview guide was used, with questions tailored to issues raised during transcription and preliminary analysis. When information was incomplete or inconsistent, additional meetings were arranged for follow up interviews, or clarity was obtained through the telephone. 

### Data Management and Analysis

Interviewers recorded data during and immediately following the interviews, and notes were combined to complete transcripts for each NPO according to the year of the interview. Data were stored in both electronic and paper format, kept under password-protected electronic files and in controlled locked cabinets for security, respectively. The names of all participating NPOs were entered into and analysed in a spreadsheet and allocated coded numbers, linked to summary data for each NPO. Transcripts for each organisation—one to three depending on the number of interviews—were analysed thematically using open coding. Themes and subthemes were generated by segmenting and aggregating codes and were verified through directed content analysis. Pseudonyms were used to protect the identity of participants during data presentation. Member checking was used to ensure accuracy of data and to increase rigour. 

### Data Reduction

In this study, we made a distinction between the year the NPO was identified by the research team (between 2010 and 2012) and the year the NPO was formed as reported by the participants during data collection. The year an organisation was formed was a reflection of the point at which some form of official or symbolic launch of the organization took place and did not necessarily translate into the onset of service provision or activities, which was considered to be commencement of operations in this study. We also recorded the year the certificate of registration as a NPO was received. Founders were defined as those individuals or groups who led to the formation of the NPO, and not necessarily those who started the operations.

### Ethical Considerations

Ethical approval was obtained from the human research and ethics committee at the University of Witwatersrand, Johannesburg under study protocol clearance number M090232, as well as the health research and ethics committee in Mpumalanga Province. NPOs participated in this study as part of a serial situational analysis to create context for phases II (in-depth interviews), III (focused group discussions) and IV (quantitative analysis) of the parent study. Participation of NPOs in the study was voluntary, and informed consent, approved by the ethics committees, was obtained verbally prior to data collection from all interviewees participating on behalf of their organisations. The consent process was documented in the interview transcripts and field notes along with other details regarding the participants. Interviews were conducted in the preferred language of the interviewees.

## Results

**Figure 1:**
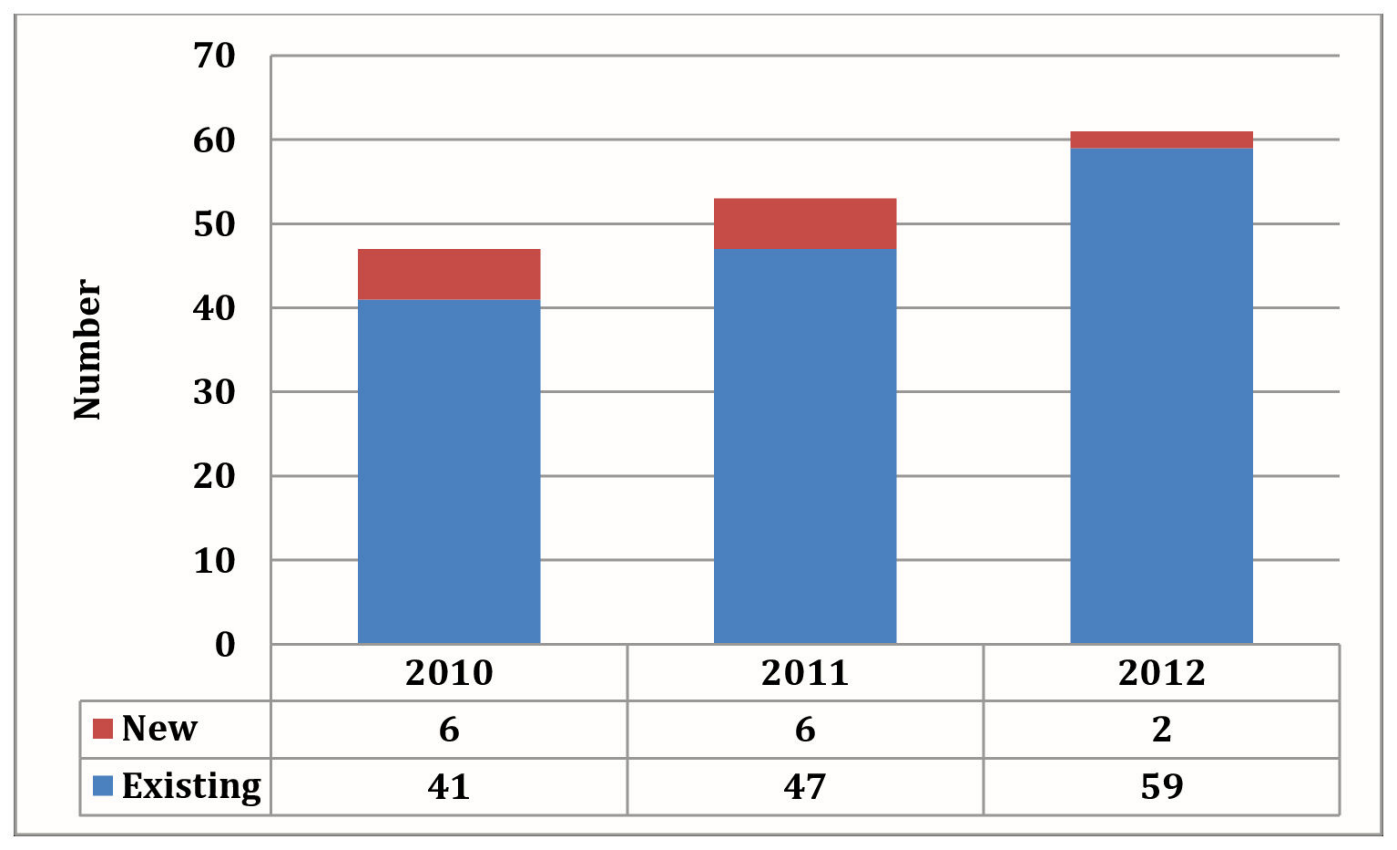
New and existing NPOs by year (N=37 in 2010, 43 in 2011 and 61 in 2012).

We interviewed 61 organizations between 2010 and 2012. In 2009, a list provided to the study team by government included 22 organisations, and the remainder were identified through networking and snowballing processes. In 2010, we identified 37 NPOs, rising to 45 in 2011 and 61 in 2012. However, some of the NPOs identified in the follow up years were not newly established, and had been missed from previous inventories. We therefore retrospectively reconstructed the actual number of existing, newly formed and inactive NPOs for each year. This is represented graphically in Figure 1. During this period, 14 new NPOs were formed and four became inactive or closed down, giving an overall growth in NPOs of 22% from the 2010 baseline. Only one of the NPOs was categorised as an NGO, implementing services throughout the province, although a smaller CBO branch was formed for the purposes of its operations. The remainder were all CBOs. 

Thematic areas identified during analysis were NPO founders, formation, service delivery, and registration.

### Founders

There was a gendered pattern in the characteristics of persons founding NPOs – 42/61 (69%) were women. One of the founders explained, ‘There are few men in this work. Men have trouble touching hearts, and are looked down upon in the community. So it’s hard to keep them [as caregivers]’. Many founders described themselves in one way or another as caregivers, some having cared for family members who later died. Two women founders were identified as church pastors, two others as wives of church pastors, and four more as active members of their church. Of the 19 (31%) males reported as founders of organizations, four were church pastors and gave religious motivations for starting the NPO. Also among the founders were four retired nurses (three female, one female), and one active professional nurse (female). Four of the founders, two women and two men, identified themselves as businesspersons with some entrepreneurial skills. There was also a policeman, a teacher, a social worker, a youth development agent, and a female founder who was illiterate. In the majority of cases, the founder was easily singled out. However, in a number of instances (5/60) the founding membership was presented as a joint team effort without any particular hierarchy. Typically in such cases, three to five women reportedly formed an organization, although the idea may have come from one of them. These so-called ‘care groups’ of women have their origins in a health sector movement beginning in the 1980s which sought to mobilise community women around and health, environment and poverty issues [19]. Five older women who had been part of a care group formed NPO 44. However, they abandoned their work when the NPO was finally formed because, as they indicated, they could not read or write. Therefore, although the care group founded the organization, they decided to hand their legacy over to a younger generation of CCWs. Two of the older women were later invited to form part of the board of directors for the NPO. In five of the 19 NPOs where men were declared as founders, they were in fact requested by a care group of women to help steer the NPO on their behalf.

### Formation

Although hampered by lack of data on NPOs that may have closed down prior to 2010, Figure 2 suggests a steady rise in the number of new NPOs formed in the course of the 20-year period between 1990 and 2010. There was only one NPO known to have been officially launched before 1996, NPO 27, which previously acted as an umbrella body of NPOs in the study area (and which subsequently lost its oversight status due to misappropriation of funds). There was a sharp rise in new NPOs between 1996 and 2000, expanding by 25% to 16 newly-formed NPOs between 2001 and 2005. There was a doubling in the number of new organizations between the periods 2006 to 2010. 

**Figure 2:**
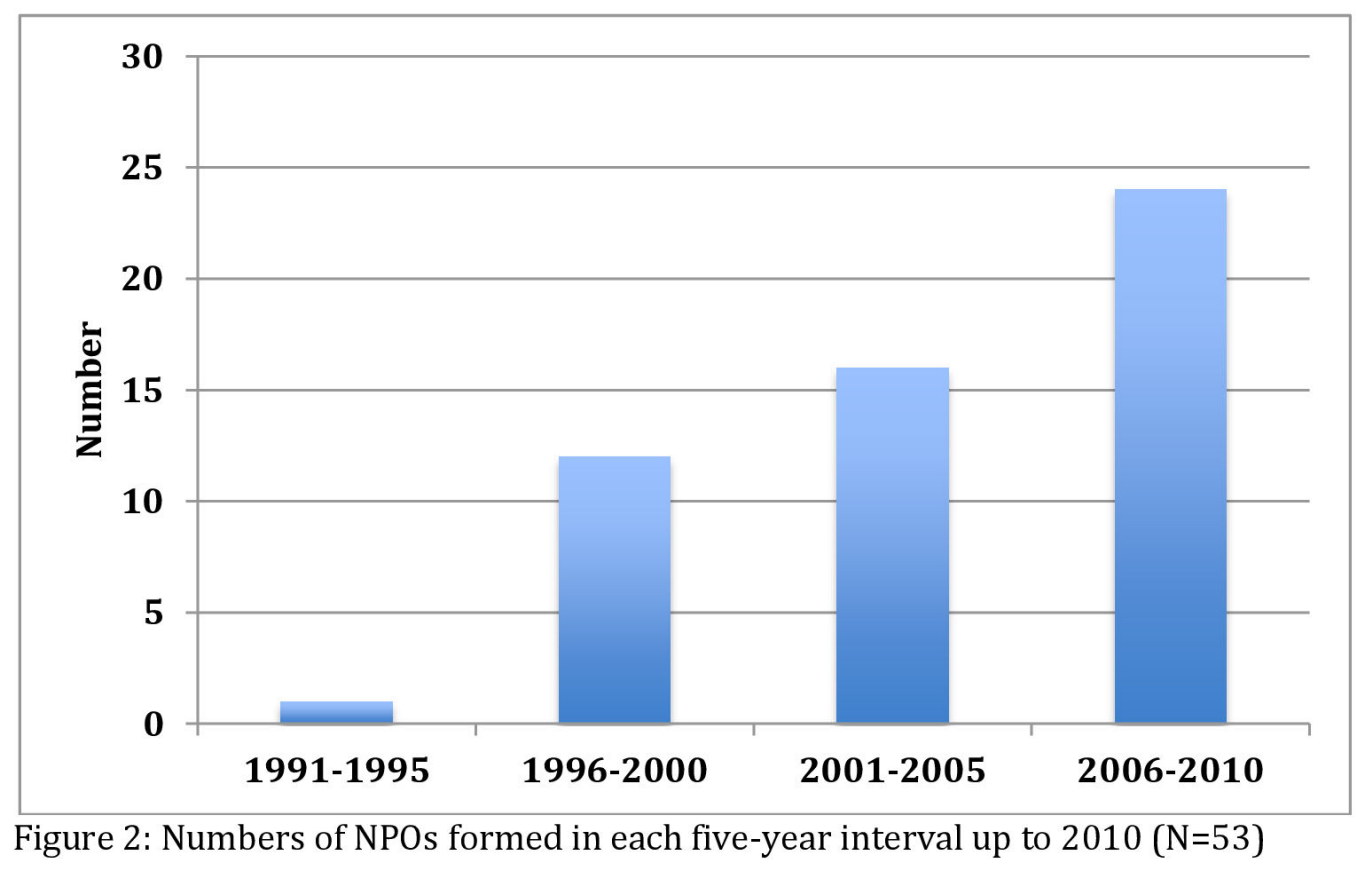
Numbers of NPOs formed in each five-year interval up to 2010 (N=53).

Many of the new NPOs were offshoots of older NPOs. At least 16 NPOs are known to have emerged out of *first tier* care groups of older women formed in the 1980s. The *second tier* care groups were composed of relatively younger women working in the same or nearby villages under the same NPO, who had decided to start NPOs in their neighbourhoods— mainly due to long travel distances. Furthermore, an additional seven parent NPOs, all formed between 1997 and 2001, gave birth to a total of 15 other organisations. Two of these parent NPOs, formed in 2000 and 2001, single-handedly led to the formation of eight (four each) more recent NPOs, all of which are now operating autonomously.

### Service Delivery

More often than not, NPOs indicated providing services long before founders declared an actual launch or structural formation. Of the six NPOs formed in 2010, four had already begun operations long before 2010, ranging from 1994 to 2003. One of the organisations reportedly formed in 2011 had actually been running operations since the year 1997. NPO 033 began providing services in 1982, but as a care group. 

### Registration

**Figure 3:**
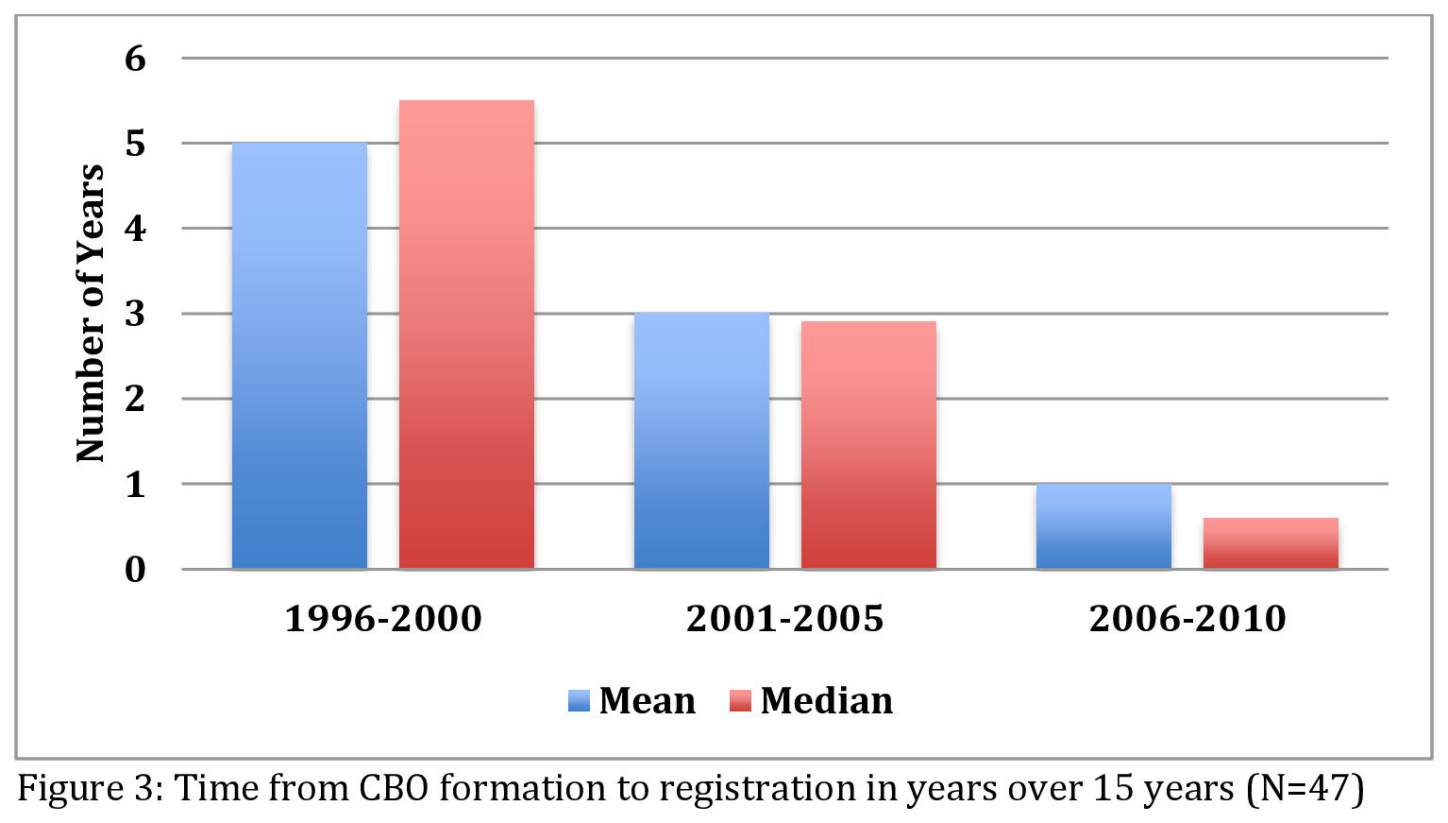
Time from NPO formation to registration in years over 15 years (N=47).

A critical milestone for NPOs was to obtain a NPO registration certificate with the government, which included a renewal process. In this study, we found that 50/61 (82%) NPOs were registered. In earlier years, all NPOs were first formed, and then later registered. Without a registration certificate, NPOs are not recognized as official structures and cannot apply for financial support. Six of the unregistered NPOs were relatively new among the eight formed in 2011 and 2012. Four NPOs formed between 2006 and 2010 were not registered, as well as one formed in 2002 that completed ten years without registration. The average time taken from formation to registration has been decreasing over the years. Figure 3 depicts a consistent trend of decline in the number of years taken to register over a 15-year period. 

**Figure 4:**
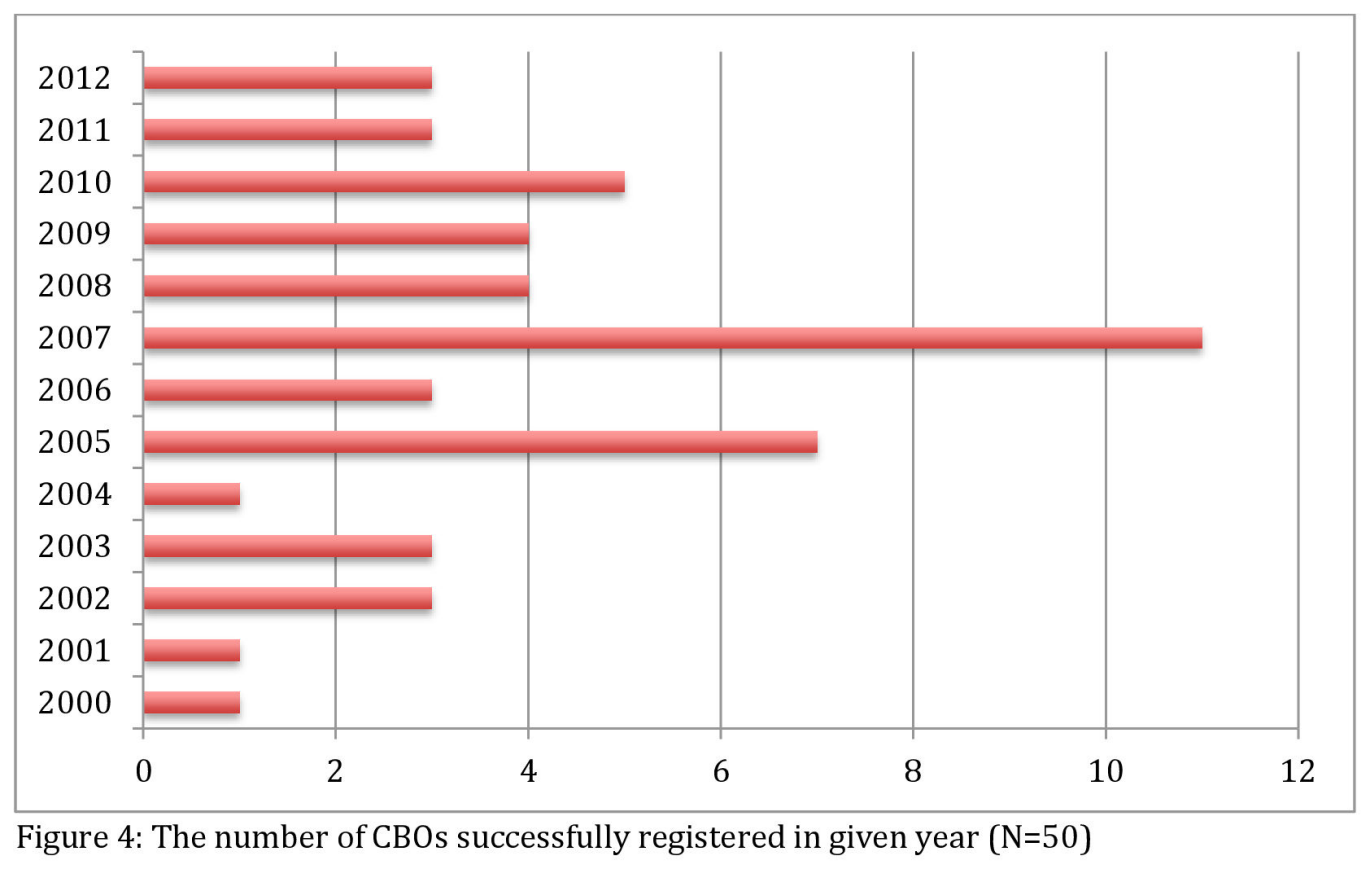
The number of NPOs successfully registered in given year (N=50).

Eight NPOs were successfully registered among the 14 most recently formed as opposed to the three-year period between 1998 and 2000 when only one out of 12 NPOs was able to register. As shown in Figure 4, few NPOs were registering in the earlier years, but these figures improved over time, indicating what may appear to be catch-up moments in 2005 (12%), 2007 (18%), and 2010 (8%). None of the NPOs formed before the year 2010 were registered before their formation. At best, four of all the registered NPOs existing prior to 2010 were formed and then registered in the same year. However, three of the NPOs formed during the study period had registered three or four years prior to their formation, a different pattern from that observed in earlier years. All three of the NPOs showing early registration share a common thread in that they originated from the same parent organisation, and establishment of their independence was supported by the same founder. In addition, their operations were already underway before their formation and registration. Therefore, this trend may not necessarily reflect a change in the NPO registration system but rather a behavioural pattern of the organisations involved.

## Discussion

This longitudinal study examined origin and foundation of NPOs in rural South Africa, all but one of which were CBOs, and there were five notable patterns observed. First, there has been a rapid increase and spread in the number of organizations which maintained a steady upward trend. Second, NPOs attained three milestones in their maturation over time, the predominant sequence being: operations, formation, and registration. NPO registration alone was neither necessary nor sufficient for founders to start operations. Third, the interval between formation and registration has been steadily decreasing over the years, a pattern possibly indicating some degree of improved efficiency in the government’s registration process. Fourth, most founders of NPOs were women and caregivers themselves, highlighting an important gendered pattern in the characteristics of people active in forming NPOs in resource-poor settings. Lastly, the reasons for starting NPOs included religious, health professional and entrepreneurial reasons or simply the desire for association. Interestingly, the growth of NPOs also emerged from lasting forms of association dating back to health sector programmes of the pre-HIV era (care groups) [19]. 

The implications of growth in an NPO sector providing health and social services documented in this study can be viewed in two ways. On the one hand, it can be viewed as a welcome development necessary to close the gap created by weak health systems in resource-poor settings, attract additional external resources and improve service delivery [1,20]. In this way, the NPO sector expansion may be a sign of unmet need being addressed through action on the ground. On the other hand, the proliferation of NPOs, can be seen as establishing an uncoordinated and unregulated system of delivery in which NPOs and their founders are given free reign to implement their operations in the community, a general situation observed in this study. Apart from registration, explicit structured systems that offered prior approval or evaluated quality of services were absent. There is the danger that poorly regulated “pluralism” of NPO service provision may increase competition and ultimately become a battle for power and access to scarce resources, with household needs and service delivery becoming secondary goals [15]. 

Some form of regulation of NPOs providing community based health and social services would thus seem necessary, although not easy to implement. The most basic of these strategies is registration, the only one reported by participating NPOs in this study. According to Bratton, governments may try to coordinate, co-opt or dissolve NPOs with tighter forms of regulation but NPOs can respond to such regulatory strategies by employing tactics to evade undue control [15]. In this study, we observed several NPO tactics that bear relevance to systems of regulation. NPOs were able to achieve their first milestone by quietly commencing operations, with neither a recognizable NPO structure nor proof of official registration as an NPO. In so doing, they kept a *low profile*, a tactic that restricted NPOs to the margins of the health care system, but adopted effectively by NPOs to avoid confrontation with government. Some degree of visibility was achieved when a NPO acquired a recognizable physical structure as their head office and underwent a public launch. NPOs cooperated with well-established NPOs, such as was seen with NPO parentage. In order for NPOs to independently enter the policy arena, and qualify for competitive fundraising, a legitimate NPO registration was an absolute requirement. As a result, the provision of NPO funding through government structures [16] may have encouraged the formation and registration of NPOs.

The patterns of formation and parent structures for NPOs suggest an internal dynamic fuelling growth in the health and social sector, which necessitates further attention in future research. In this setting, findings of the study revealed the gendered social dynamics of the NPO sector. We know that CCWs are mostly female in South Africa [21,22]. Our study found a similar pattern among founders of NPOs, who generally believed that women had a natural ability to nurture [23]. Some NPOs deliberately recruited men as leaders, apparently, to increase their legitimacy and social standing. Such gender issues, while well recognised, are seldom addressed explicitly in policy processes, and yet carry implications for the design of broader structural interventions [24,25]. 

### Limitations

Our study was not without limitations. First, data collection methods relied mainly on exploratory approaches, the findings are tentative, and we do not claim generalizability beyond the context of the study. However, to our knowledge there is no such study conducted in the country. Second, the historical view taken during data collection exposes the study to recall bias and false data. However, the follow up period over three years increased rigour and confidence in the findings, complemented by member-checking at follow-up visits to verify the validity of the data previously provided by participants. Third, there may have been NPOs that were unaccounted for, but repeated visits to the field and the networks established over three years helped minimise this risk. Also, the ability of the research to locate new organisations may have improved over time. 

## Conclusions

In conclusion, there has been rapid emergence of new NPOs in rural South Africa, and the government needs external support or intensified systems in order to identify all existing and newly formed organisations. While expansion in the NPO sector may serve to reduce existing gaps in health care system, open up employment opportunities, and create a platform for empowerment of rural women as was shown in this study, literature suggests that plurality of NPOs may grow into a source of concern. Therefore, improved systems of oversight by government may become necessary. However, a delicate balance between control and autonomy of NPOs will be needed, lest NPOs develop tactics to evade regulation. Also, the foundation of NPOs—mostly by women who are themselves caregivers driven by perceived need in their community—is a long drawn out process that undergoes several stages of development over long periods of time. Identifying appropriate roles for and forms of regulation that shape the emergence and functioning of the community based NPO health and social sector is relevant to both health care reform and rural development in general.
